# An Unexpected Reason for Isolated Foot Drop: Acute Stroke

**DOI:** 10.12669/pjms.335.13593

**Published:** 2017

**Authors:** Eylem Kuday Kaykisiz, Erden Erol Unluer

**Affiliations:** 1 Dr. Eylem Kuday Kaykisiz, MD, Department of Emergency Medicine, Bitlis State Hospital, Bitlis, Turkey; 2 Prof. Erden Erol Unluer, Department of Emergency Medicine, Usak University Faculty of Medicine, Usak, Turkey

**Keywords:** Acute weakness, Cerebral infarct, Monoparesis

## Abstract

The differential diagnosis of acute weakness (AW) in emergency departments (ED) is broad and includes both neurological and medical reasons. We describe an 81-year-old female patient with cortical infarct presenting with sudden onset isolated foot drop, which to the best of our knowledge, was the third case in English literature.

An 81-year-old female was admitted to our ED with a 12-hour history of left-sided foot drop. Her motor strength was normal throughout the upper and lower extremities, except for weakness in the left ankle and toe dorsiflexors. Other examination findings were unremarkable. Diffusion-weighted magnetic resonance imaging (DWI-MR) revealed a focal high intensity signal in the right precentral gyrus at high convexity with a cerebral infarct.

Detailed physical examinations and histories are extremely important for exact diagnosis and differentials of patients with AW. This case reminds us that a small infarct area of central nervous system may mimic peripheral nerve lesions, especially in elderly patients. Although the presentation of such complaints may play a distracting role to emergency physicians, strokes must always be taught regarding elderly patients and, if necessary, infarct areas should be confirmed with DWI-MR.

## INTRODUCTION

The differential diagnosis of acute weakness (AW) in emergency department (ED) is broad, and includes both neurological and medical reasons. AW may be originated from upper or lower motor neuron lesions, spinal cord pathologies, neuromuscular junctions and muscle diseases^1^ (Table-I).

**Table-I:** Acute weakness differentials.

*Muscular diseases*	*Neuromuscular junction diseases*	*Spinal cord diseases*	*Lower motor neuron diseases*	*Upper motor neuron diseases*
Hereditary or inflammatory myopathies, Metabolic causes such as hypokalemia, hypocalcemia, hypoglycaemia Periodic paralysis	Miyastania gravis Organophosphate intoxication Botulismus	Myelitis Multiple sclerosis Trauma Spinal infarct Acute spinal cord compression	Polymyositis Root or plexus pressure Guillain-barre syndrome Porphyria, Heavy metal intoxication Diabetes mellitus Chronic alcohol use Tick paralysis	Ischemic or hemoragical stroke Multiple sclerosis Intracranial mass or abscess

Investigating detailed familial histories, heavy metal and carbon monoxide exposures, canned food consumptions, traumas, chronic alcohol consumptions and presence of chronic diabetes mellitus (DM) may give some clues to emergency physicians (EPs). Physical examinations must be completed by examining deep tendon reflexes (DTRs), muscle tones, proximal or distal muscle strengths, and presence of fasciculation, sensory deficits or muscle atrophy. EPs should know the classic physical examination findings that differentiate between upper and lower motor neuron diseases. In contrast to upper motor neuron injuries’ increases in DTR and muscle tone and presence of pathological reflexes, neurological examinations depict decreased DTR and muscle tone, or lost pathological reflexes in addition to the presence of fasciculation, sensory deficits and muscle atrophy in lower motor neurons.

Pure motor paresis that affects one limb is rarely seen in EDs. Specifically, pure motor paresis in lower extremities, like foot drops, are first thought to be caused by traumatic damage to peroneal nerves, lumbosacral radiculopathies, spinal cord tumors or by multiple sclerosis. Foot drop is a symptom that is described as weakness or complete absence of dorsiflexion of ankles and toes. In addition to peripheral nerve lesions, non-neurological causes like acute arterial occlusion or aortic dissection, and rarely seen neurological causes, like strokes, should be kept in mind for differentials.

We describe an 81-year-old female patient with cortical infarct who presented with sudden onset isolated foot drop that easily interfered with peripheric nerve lesions. To our knowledge, this was only the third case of an isolated foot drop in a stroke patient in English literature.

## CASE REPORT

**Fig.1:**
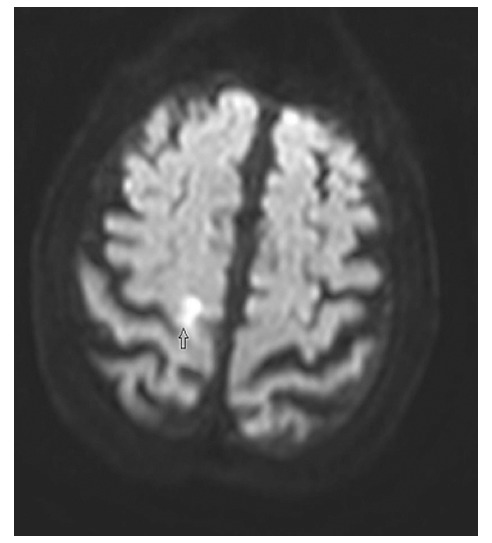
Diffusion-weighted magnetic resonance imaging of the patient revealed a focal high intensity signal in the right precentral gyrus at the high convexity area of the brain cortex.

An 81-year-old female admitted to our ED presented with a 12-hour history of left-sided foot drop. The patient’s past medical history was unremarkable. She denied any recent trauma to the left leg or lumbosacral area. She did not describe headache, lumbago, visual changes, fever, dizziness or bowel or bladder problems. On her detailed examination, she was well-oriented and cooperated, exhibiting no signs of cranial nerve function disorders, such as facial palsy, dysphagia or dysarthria. Her motor strength was normal throughout her upper and lower extremities, except for weakness in the left ankle and toe dorsiflexors. Distal pulses and sensations were intact in all extremities. No pathological reflexes were found bilaterally. The DTRs were normoactive in all extremities. Her sensory perceptions of touch, vibration and joint position were normal. Cerebellar function tests were negative and there were no signs of ataxia. Her straight leg-raising test was normal and no tenderness occurred in the lumbosacral area. Her electrocardiogram was noted as normal with sinus rhythms. Laboratory values, including complete blood count and biochemistry, were within normal ranges. Brain computerized tomography without contrast (CT) revealed normal findings. DWI-MR revealed a high intensity signal in the right precentral gyrus at the high convexity area (Fig.1) with apparent reduced diffusion coefficient in the same location. The patient consulted with a neurologist and was hospitalized. There was no progress or decline in follow-ups, and the patient was discharged with an acetyl salicylic acid treatment.

## DISCUSSION

Foot drop can be seen in any disease that causes damage to the innervating motor neurons of the dorsiflexor muscles of the ankle and toe. The most common reason for foot drop is compression of the peroneal nerve around the fibular head. Other reasons are peripheral neuropathy, peripheral nerve damage, leg compartment syndrome, and systemic diseases, such as vasculitis, DM, and connective tissue diseases.^2^ However, neurologic findings for these disorders show slower disease tempo and bilateral presence in contrast to our patient. Based on the patient’s history and physical examination, we considered central nervous system etiologies, including brain tumor, multiple sclerosis, hemorrhagic contusion from gunshot wound, head trauma and brain abscess, as opposed to peripheric ones.^3^-^5^ Most foot drop cases present with upper motor neuron signs, such as paresthesia, hyperreflexia, ankle clonus or Babinski signs. Only a limited number of cases have been described as isolated foot drops caused by central etiologies.^3^,^5^ There have only been two previous reports on isolated foot drops related to strokes in English literature.^6^,^7^ Diagnosis is usually delayed because peripheral lesions are considered in such cases because of atypical presentation. The somatotopic ankle and toe topography in the brain has previously been established in parasagittal regions by electro stimulation localization.^8^ It is concluded that cortical infarcts were more likely to cause isolated foot drop. The lesion in our case is consisted with the alleged motor homunculus in the brain.

According to the literature, the first 72 hours in stroke management is important and can be associated with the infarct progression.^9^ In omission of the patient presenting with an isolated symptom that in consisted with the cerebral infarct such as foot drop, it may results with the additional symptoms and progress of stroke because of the delayed treatment.

## CONCLUSION

It should not be forgotten that if the detailed physical examination and history are not taken in patients with isolated sudden foot drop, the diagnosis of the patient will easily be mistakenly made as a peripheral lesion and the life quality of the patient may deteriorate due to the progression of the infarct. Isolated foot drop is a condition that should be kept in mind in patients with monoparesis and if necessary, this infarct area should be confirmed with DWI-MR.

### Authors’ Contribution

**EKK** did data collection and manuscript writing.

**EEU** did review and final approval of manuscript.
